# The Advantages and Disadvantages of Specialism

**Published:** 1894-01

**Authors:** Reuen Thomas

**Affiliations:** Brookline, Mass.


### THE ADVANTAGES AND DISADVANTAGES OF SPECIALISM.[1]

BY REUEN THOMAS, D.D. PH.D., BROOKLINE, MASS.

Mr. President and Gentlemen,—Ever since I accepted your invitation to be present at this meeting I have been in a state of constant wonderment at my own audacity. I have no kind of experience or ability which would qualify me to speak on dentistry. The moment I began to do that my position would be not dissimilar to that of the clown in the circus. If you have invited me here

---

[1] An address delivered before the American Academy of Dental Science at its annual meeting, Boston, November 15, 1893.

with the idea of aiding your digestion by increasing your laughter, I am afraid you will fail of your purpose, for I do not intend to speak on dentistry. So long as I do not commit myself to speech on that painful theme, I may get credit for knowing something about it. The probability that some of the learned gentlemen here present would not be able to follow me if I should venture upon the scientific treatment of the theme is one of the reasons why I forbear. When I tell you that my definition of a tooth would be about like this: "A tooth is a structure in which a process of the epiblastic layer—the buccal epithelium—grows into the subjacent dermis, and, assuming a cup-like form (with the concavity of the cup turned away from the epithelial surface of the mouth), a dermal papilla rises into the cup;" that the definition of a tooth would be something like that, and that the whole of the address would be consistent, both in language and lucidity, with this definition, I have no doubt of your congratulating yourselves that no such process stretches before your vision.

Modestly discarding all idea of mystifying this learned body by an essay on dentistry too scientific for general comprehension, I asked myself whether it might not be expected that I should speak, as one might say, anthropologically, and treat men as if they were teeth. Under that idea some men would be regarded as incisors, some as molars, some as sound, and some as decayed. Under these headings we might possibly make a new and accurate division of the human race. These and other dental suggestions would lead us to such inferences as these: that there are some men we could do without and be all the happier; that as false teeth have sometimes a more pearly and regular appearance than true teeth, so also false men are sometimes even better looking outwardly than true men, and that Iago and Mephistopheles in all probability were most fascinating gentlemen. Along that line of remark we might find much that was interesting and profitable.

It seemed highly probable that you might want to know something of the relation of dentistry to theology. But the more I mused on that theme the more difficult it seemed to make the connection. That there is such a connection I have no manner of doubt. But the immediateness of the relation is not easy to discover. Jacob's ladder had its foot on the earth and its top seemingly in the heavens. Between the lowest and the highest round of that ladder there was a connection, and so, I suppose it is true that everything is connected with everything else. We know that teeth have a very important relation to food, and that food has a very close relation to digestion, and that digestion may become indigestion, and that under an attack of indigestion a clergyman might proclaim a very dark-hued and Satanic theology, and unpityingly and remorselessly send all his hearers into the Inferno so vividly imagined by Dante. So that there can be no doubt that dentistry has a relation to theology, and bad teeth may account for much bad theology, as also for the bad delivery of good theology. No one can question that there is a very manifest association between dentistry and speech. An indistinct and unintelligible utterance will spoil the effect of the most thoughtful speech, or the most intelligent sermon. The responsibility of dentists for much bad theology and poor preaching may therefore be greater than even the most thoughtful members of the profession have ever in their most serious moments assumed to be the case. If only it were possible to make a dentist's chair easy and attractive, so that it did not associate itself with pain, the most timid and nervous men and women would seek it as a matter of course, the men to be made useful and the women to be made beautiful forever. I do not mean to suggest that the speech of the woman part of mankind is not sufficiently distinct and emphatic as it is. But man is a speaking animal as well as an eating animal, and a skilful, scientific, humane dentist may be of as much service to all men whose profession involves public speech as is the first-class professor of elocution. For the ability which a tooth has of punishing or serving its owner is among the things that are remarkable. A tooth can be as annoying as an Irish Bridget, as ingenious in inflicting pain as a Mediæval Inquisitor, or as serviceable to its master as Caleb Balderston in Scott's "Bride of Lammermoor."

But leaving all this preliminary skirmishing, confronted as I am with gentlemen who are specialists, I wish to offer a few thoughts on "Specialism: its advantages and its dangers." Dentistry has attained to the dignity of a profession. By and by a volume will be published, I do not doubt, entitled "Great Dentists," as now we have volumes on "Great Surgeons," "Great Lawyers," "Great Divines." But the time has not yet come for such a volume, as the profession is comparatively new. As a writer in the "Encyclopædia Britannica" puts it, "For long, dentistry was practised to a large extent as a superadded means of livelihood by persons engaged in some other pursuit, and without any professional education whatever. The blacksmith, the barber, the watchmaker, and others of the same class were the dentists of every village and country town; while even in some of our larger cities dentists of the kind were till lately to be found practising under the very shadow of our universities and public schools." These words refer primarily to England, but they sufficiently well fit the past condition of things on this side of the ocean, if the speech of the men of seventy years and upward is to be taken as reliable. In our days it is not considered so respectable for a man to be a general practitioner as it is to be a specialist. The time has gone by when one and the same man taught classics and mathematics. The professor of Latin seldom ventures nowadays in any but a third-class academy or school to be also the professor of Greek. Every man has to be more or less of a specialist. The definition of a learned man or a scholarly man in our day has changed from what it was in the days of our grandfathers. Then a learned man was a man who professed to know all that was knowable of most everything. He was a walking encyclopædia, or, as was said of Macaulay, "a book in breeches." Now the encyclopædic man is generally regarded as a charlatan. A learned man in our day is a man who knows everything of something and something of everything. Some of his knowledge must be microscopic, but most of this knowledge telescopic. He is minutely acquainted with some one thing and generally acquainted with many things.

I assume that the gentlemen here present know everything that is to be known about teeth. And yet I doubt it. In order to know everything that is knowable about teeth, is it not necessary that all and everything which is preliminary to the growth of a tooth should be known? It is certain that teeth are associated with the whole animal organism, and that the whole of the organism contributes to them, so much so that the reason why you and I have the teeth that characterize us is not satisfactorily accounted for by simply examining the teeth. At the back of the teeth is the blood in the veins, and at the back of that one knows not how many rogues and rascals in our ancestry until we get back (as one gentlemen I talked with the other day assured me he did) to William the Conqueror.

In order to give a full and particular account of anything belonging to any individual man, it is necessary to know vastly more than is revealed in the man's own individuality. A certain English nobleman, recently deceased, was what is called a kleptomaniac. He had everything that heart could wish, much more of wealth than any sane or insane man could legitimately use, and yet if he were left by himself for a while he would be like that politician who the first time he found himself in Washington, contrary to all his former propensities, had an overmastering desire to steal something. So this English nobleman, richer in money than Crœsus himself, had in his blood the persistent tendency to steal. How had it got there? Had it no relation to the fact that in days long ago his ancestors used to go on foraging expeditions and drive off the cattle of their weaker neighbors?

Now, if a learned man is a man who knows everything about some one thing, a learned dentist should be a dentist who knows everything that is knowable about teeth. But in order to do this he must know something about anatomy and physiology, and much else besides. For anatomy and physiology are associated with psychology and mental philosophy generally. The influence of mind on matter is only beginning to be appreciated. We are on the edge of a realm of knowledge which is as certainly hidden from us now, or revealed to us only suggestively—as much hidden as was the realm of the infinitely little before the invention of the microscope, or the realm of the infinitely great before the invention of the telescope.

Now, the tendency of specialism is to take the telescope out of a man's hand and leave him only with a microscope. The tendency is to imprison him within the limit of his specialism—to bring his mind under the .power of the ideas and laws of that specialty which he is constantly studying. The mind of the specialist has a tendency to conform to the thing about which it is persistently and continuously occupied, to take its tone, its color, its mode of vision, the way in which it looks at things from the particular region to which it bends its attention with most interest and with daily recurring constancy. The mathematician gradually loses all power of estimating the value of anything which cannot be mathematically proved. Unless by studies not mathematical he keeps his mind fresh and free, some of the most fascinating departments of knowledge will gradually lose their influence over him and he will be as mentally enslaved as the man who, looking at a most suggestive work of art full of life and expression, could make no remark but one. The size of the canvas led him to exclaim, "I wonder how many pounds of paint it took to produce that thing?" So, also, the biologist, the geologist, and other ologists, confining themselves to their specialty, gradually get their minds trained down to the conditions under which they are daily working, and come into an ability of appreciating only one kind of evidence. Illustrations of this abound. There was never a more convincing instance of the way in which the mind of a very eminent and distinguished man comes to settle into the mould of its specialty than when some years since Professor Tyndall asked of certain London divines that the question whether God answered prayer should be submitted to a practical test suggested by himself. The test was that the London Hospital should be divided into two sections. The church should offer up its prayer for the sick on behalf of the people in the wards on one side of the hospital, and not those on the other side. The result of the experiment should decide the question. Now, it is scarcely credible that such a suggestion, from the mind of such a man, could have been made seriously. Both God and all good praying people must have accepted as reasonable and intelligent Professor Tyndall's suggestion before it could have been made. To begin with, Tyndall's idea of prayer was not Christian. It was so ignorant an idea of what prayer is that a well-instructed Sunday-school child would see the defect in it. That Tyndall was serious there can be no doubt. The fact shows one of the dangers of our nineteenth century specialism, and how it reduces the minds of even superior men to the level of the particular branch of science in which they are constantly working, as the dyer's hand takes the color of the dye in which he puts it. That is why in that wonderful "Journal of Amiel," next to the thoughts of Pascal one of the most suggestive books of its kind ever issued, the writer says, "Science does not make men, but only entities and abstractions." Scientific men are so given to analysis that many of them live in a dissecting-room for the period of their natural life. We may analyze a rose till we have no rose. We may dissect a human body till we have no body. Too much concentration tends to fix the mind on parts to the neglect of the whole. That every part suggests a whole, and needs a whole to explain it, is a fact of which we must never lose sight. That is what Tennyson is trying to say when he plucks the little flower out of the crannied wall:

> "Flower in the crannied wall,
> I pluck you out of the crannies;
> Hold you here, root and all in my hand,
> Little flower, but if I could understand
> What you are, root and all, and all in all,
> I should know what God and man is."

Specialism has, then, the advantage of giving a minute knowledge of some one part of the human organism, or of some confined region of science. The man who gives exclusive attention to the eye eventually arrives at a knowledge of all abnormal conditions to which that organ is liable, gets to discern every symptom and sign of disease, and to acquire all the scientific knowledge and skill which up to date belongs to his particular specialty. So with the ear, so with the throat, so with the brain,—a much more complicated specialty than the others I have named. So with every other organ and region of the body. And these specialists are the men to consult if you can afford to pay them their high fees, and if you are intent on commanding the best assistance which science can offer. Specialism is a distinct advance upon generalism. Not one word other than a word of gratitude to that Providence which is always moving the race onward towards something higher and better have we to offer in regard to it. Its helpfulness is often wonderful, its advantages are beyond question. So far as the general public is concerned, specialism is an immense gain.

But I am thinking of the effect of specialism on the specialists themselves. The question whether we are not in our generation growing a large crop of men who are learnedly narrow, and that because they are confined to too microscopic work, to research into detail rather than to research on the broad continents of knowledge, is a question which cannot be waved aside as if it were not worth the asking. There are laws of mind which are unalterable. The mind conforms itself to what it persistently studies. The laws of that branch of science control in its judgments. They determine its modes and methods of thinking. Their tendency is to materialize the mind, and to compel it to think on all subjects mental and moral as if there were no other laws in the universe than material laws. So far as his thinking goes, a man is apt to see everything through the spectacles that have insensibly come over his eyes from the specialty with which he is persistently occupied.

As an illustration of what I mean, let us take the work of a modern man of eminence,—a man of whose goodness and greatness those who know him personally have no doubt. I mean Professor Henry Drummond, whose lectures at the Lowell Institute here in Boston commanded so much attention. The book which made him famous is entitled "Natural Law in the Spiritual World." "None," says Professor Drummond, "except those who have passed through it can appreciate the radical nature of the change wrought by science in the whole mental attitude of its disciples." And he goes on to teach with much wisdom and learning, and with that charming style of his which makes it so easy and so delightful to read him, how the thought of evolution permeates everything. He seems to be quite unconscious of assuming very much more than he can prove, in writing his book on the principle that "natural law," by which he means the law which is revealed in material nature and deduced from it, is identical with spiritual law,—the law of mind, and will, and imagination. His book is constructed on the assumption that natural law and spiritual law are identical. He assumes that the same laws which govern vegetable and animal life govern mind. I venture to say that he assumes it without proof. Indeed, we may say with much of positiveness that it is utterly impossible that the laws of the spiritual world, considered as a whole, can be identical with the laws of the natural world. The simple facts of free will and moral responsibility are themselves enough to create a radical difference between the two worlds. I know from personal communication with Professor Drummond, that if he had to issue his book now for the first time, that it would come from the press simply as an analogy and not as an argument,—simply as supplying illustrations which are wonderful and helpful. It is only fair to say that much. But it is an illustration of the fact, of which there can be little doubt, that the devotion of the mind to scientific pursuits is apt to engender a certain outlook on the universe, which, if not corrected by other considerations, may find it difficult to recognize the signs of the supernatural in nature and in history.

Now, if a man like Professor Drummond, who is a remarkably open-minded man, can fall into this error of assuming that material law is the measure of all law, what are we to expect from men of a much lower type than he? Is there not ground enough and reason enough to say to all specialists, Beware of assuming that all the laws of the universe are to be interpreted by the laws you find working within the narrow area to which your study and observation are confined? Is there not a necessity to say to men who are specialists, Be sure you do not live and move and have your being within the limits of your specialism? Correct the tendency which your specialism creates to learned narrowness by taking excursions into all kindred departments of knowledge. It is marvellous how a man may all but destroy his ability of enjoyment in the region of his higher faculties—the poetical and historical faculties of his soul—by a servile attention to things that are minute and simply external. I was reading not long since, in a book by Dr. Birkbeck Hill, of Oxford, the following incident, which is by no means a bad illustration of what I mean. "A friend of mine in this University told me that before he had come up to Oxford he had read Virgil's 'Æneid' through with great delight. In preparing for the examination known as 'Moderations,' he was taken through it by his tutor once more, who treated Virgil not as a great poet, but as a convenient instrument of instruction in the niceties of grammar. Under the guidance of this teacher—

> 'One whose hand,
> Like the base Indian, threw a pearl away
> Richer than all his tribe'—

my friend gained his first class and lost forever his enjoyment of the Æneid.

"The man who would use a great poet for beating grammar into a boy, who would parse 'Hamlet' and analyze the 'Paradise Lost,' would not for one moment hesitate to botanize upon his mother's grave."

With all the advantages of specialism, and they are manifestly many, it is necessary to recognize that a man's specialism may so tyrannize over him as to drag him down out of the freedom and largeness of his manhood into being simply an oculist, or an aurist, or a dentist,—the laws of specialism so controlling the man that he views everything on earth, above the earth, and under the earth as a mere mechanic, and not as an artist. Even a man who is technically an artist and paints pictures may and often does become a mere materialist, a-copyist of the externals of nature, with no imagination and no spiritual faculty whatever. The Greeks themselves, when they had attained to the highest competency in sculpture ever reached in the history of the human race, seemed to lose all ability except the one ability which has immortalized them. They had studied the human body and the art of expressing it in marble until they lost all sense of modesty and decency, all ability of appreciating the moral and intellectual, so that a Pericles was a nuisance to them, and for Socrates there was nothing better than the cup whose content was death.

While, therefore, we pride ourselves on the wonderful advance we have made in dentistry, surgery, and other departments of knowledge and achievement during the last half-century, there is a necessity for reminding ourselves of the tendency of an age of specialists to produce men in large numbers who are illustrations of learned narrowness, men whose minds are warped, sectarianized, and materialized,—men whose world is a very much smaller world than it would have been if they had given serious heed to other studies than the bread-and-butter studies of life.

Moreover, it ought to correct our tendency to conceit and selfcongratulation when we remember that with all our advance there are some things—yes, many things—which animals and even insects can do better than we can do. In their special line, bees and ants are capable of giving points to men. The business aptitude of ants is as great or greater than that of men. The science of government in the United States appears like the veriest tyroism when we go to the bees. Our rebuke of laziness and do-nothingism is feeble indeed compared with the practical indignation shown by bees to drones. "The more I know of dogs," says Madame de Staël, "the less I believe in men." We might add: the more I study insects, birds, and animals, the less I pride myself upon my mechanical superiority and my genius as a specialist. There is scarcely anything that man can do along the lines of these modern specialisms, however wonderful it seems, that cannot be eclipsed by something which some insect or bird or animal can do.

Man's superiority is in the connecting and associative faculties of his mind. His ability of connecting his knowledge with all other knowledge; his ability of large discourse, looking before and after,—yes, and we may add above and below. He connects this earth with that multitude of earths which we call the planetary and stellar systems, and thus realizes that he is not only a citizen of the world, but has an inheritance in a universe. Not only has he world faculties, but universe faculties. He has little use for them at present, but every germinal unused faculty has a promise and prophecy in it. I think it was that very extraordinary man, the late Professor Sterling Maxwell, of Cambridge University, England, who suggested to us that in our nature there are latent faculties for which, in this present world, we have no use. Apart from their existence, it is, according to Professor Maxwell, very difficult to account for certain peculiarities in the higher faculties of men. This tendency of men to associate the known with that which the known suggests,—to associate the seen with the unseen,—a tendency found in all the highest natures, puts man in the scale of being far above every other creature occupying this earth. If this competency is distinctive of man, it indicates that his manhood consists not in anything in which he is like other creatures, but in that in which he is unlike them. Manliness is something quite distinct from animalness. It is only as we conquer and subordinate the animalness in ourselves that we develop the manliness.

We have often been reminded that there is such a thing as *professional pride.* I think such pride to be good and worthy. Do we not owe to the profession to which we belong such character and conduct as will add to its worth, respectability, and dignity? We hold the reputation of other men in our keeping, as well as our own. Every profession has in it men who are unworthy of it, men who regard it simply as a business by which to earn bread and butter. Doubtless there are such vulgar souls in your profession, as there are in mine. I say "vulgar souls," using the word "vulgar" in the way in which it is used by that artist in the use of language, John Ruskin. "Vulgarity," he writes, "consists in a deadness of the heart and body, gentlemanliness being another word for an intense humanity." Vulgarity shows itself in inability to feel or conceive noble character or emotion. This is its essentially pure and most fatal form.

There is no reason in itself why the profession to which gentlemen here present belong should not give time and opportunity and stimulus for nobler studies than those immediately suggested by a narrow view of its area. There is no reason why in it, as in surgery and medicine, there should not be developed a professional pride. But it is well for us all sometimes to face the great facts of life and interrogate them. Whatever our business in life be, it may be dignified or undignified. We may be artists or mere mechanics. As Gladstone says, "Our life may be food to us, or it may, if we will have it so, be poison." Every profession has its temptations, mine and yours. You know what yours are better than I can tell you. To cherish professional pride is helpful to triumphing over these temptations. An organization like this, and everything else which lends dignity to the profession, is to be fostered as good. If it were to be assumed that your profession had already attained to the utmost of its excellency, such an assumption would only indicate how narrow were the limits within which your profession moves.

The time will come when a visit to the dentist will have no more terror in it than a visit to the physician, who simply feels your pulse and looks at your tongue and asks you the usual questions, and then gives a bottle of sugar-coated-pills. It is this perpetual striving after excellence on the one hand or the want of it on the other which, more than the original difference of gifts (certain and great as that difference may be), contributes to bring about the differences we observe in the works and characters of men. It becomes us never to forget that while professional and other men seem to be doing so many things, each and all are doing one thing,—the building of character. No man living can escape that. Every day and hour that is going on. That most popular of all professors in the Universities of Scotland, Professor John Stuart Blackie, of Edinburgh, may be a trifle dogmatic when he says, "The merely professional man is always a narrow man; worse than that, he is in a sense an artificial man, a creature of technicalities and specialties, removed equally from the broad truth of nature and from the healthy influence of human converse.

"But if a man will fix his mind on merely professional study, and can find no room for general culture in his soul, let him be told that no professional studies, however complete, can teach a man the whole of his profession; that the most exacting professional drill will omit to teach him the most interesting and most important part of his own business,—that part, namely, where the specialty of the profession comes into contact with the generality of human notions and human sympathies. Universal experience, accordingly, has proved that the general scholar, however apparently inferior at the first start, will, in the long run, beat the special man on his own favorite ground."

Such an opinion of such a man justifies, I think, my choice of a theme on which to offer the ideas I have been able to suggest,—the advantages and dangers of specialism. A young girl who had an offer of marriage she wished to accept submitted the matter to her father, who did not wish to have her vacate his home, and so advised her against matrimony, using as an argument the quotation from St. Paul, "Those who marry do well; but they who do not, do better." "Well," said the damsel, "I love to do well; let those do better who can." In accepting your invitation, I thought that perhaps I was doing well; probably, by this time, some of you will be thinking that if I had declined, I should have done better. However, I may shield myself under the example of that apostle who, when the lame man at the beautiful gate of the temple asked an alms, replied, "Silver and gold have I none; but such as I have, give I thee." I hope that whenever I meet any of the gentlemen present, it may be under conditions when I shall have as little pain and as much pleasure as on this festive occasion.

